# Treatment outcomes and comparative survival analysis of intraductal carcinoma of the prostate

**DOI:** 10.1007/s11255-025-04485-3

**Published:** 2025-04-05

**Authors:** Taylor Stamey, Kristen Armel, Andrew W. Ju, Shoujun Chen, Musharraf Navaid, Arjun Bhatt, Michael C. Larkins

**Affiliations:** 1 Brody School of Medicine (BSOM), East Carolina University (ECU), 600 Moye Blvd, Greenville, NC 27834 USA; 2 Department of Radiation Oncology, ECU, Greenville, NC USA; 3 Department of Pathology & Laboratory Medicine, ECU, Greenville, NC USA; 4 Division of Hematology/Oncology, Department of Internal Medicine, ECU, Greenville, NC USA; 5 Department of Emergency Medicine, Boonshoft School of Medicine at Wright State University, 2555 University Blvd, Fairborn, OH USA

**Keywords:** Prostate cancer, Intraductal prostate histology, Cancer epidemiology, Chemotherapy, Radiotherapy, Surgical oncology

## Abstract

**Purpose:**

Intraductal carcinoma of the prostate is a rare subset of prostate cancer, for which no consensus treatment guidelines exist. We seek to investigate treatment and survival outcomes for IDC-P in the context of current NCCN guidelines.

**Methods:**

The Surveillance, Epidemiology, and End Results (SEER) database was queried to identify patients with intraductal carcinoma of the prostate diagnosed between 2000 and 2020. Cox regression analysis and log-rank comparisons of both overall and cause-specific survival over 5- and 10-year timeframes were conducted.

**Results:**

945 patients were identified. Cox regression analysis demonstrated treatment with unimodal surgery (hazard ratio (HR) = 3.70, *p* = 0.005) was associated with decreased 10-year cause-specific survival, while unimodal treatment with radiotherapy was associated with decreased 5- and 10-year overall survival (HR = 2.14, *p* = 0.025; HR = 2.16, *p* = 0.005, respectively). Univariate survival subanalysis of treatment regimens demonstrated decreased 5-year cause-specific (*p* = 0.004) and overall (*p* = 0.019) survival among patients that received only radiotherapy as treatment. Radical prostatectomy alone was non-inferior to radical prostatectomy with adjuvant radiotherapy in the context of 10-year overall survival (90% vs 80%; *p* = 0.58).

**Conclusion:**

Differences in both 5- and 10-year overall survival and cause-specific survival were present among patients diagnosed with IDC-P. Treatment with unimodal radiotherapy among patients with IDC-P was associated with decreased survival compared to treatment with radical prostatectomy ± adjuvant radiotherapy, while radical prostatectomy alone was non-inferior to radical prostatectomy with adjuvant radiotherapy. Further research into the risk stratification and optimal treatment of these patients is warranted.

## Introduction

Intraductal carcinoma of the prostate (IDC-P) is a rare prostatic neoplasm found in approximately 3% of all prostate biopsies [1]. Considered an aggressive type of prostate, it is associated with high-grade and high-volume, invasive prostate cancer in general [2]. It is relatively new in terms of accepted tumors of the urinary tract and male genital organs, having been added to the World Health Organization classification in 2016 [3]. Comparatively, ductal prostate carcinomas account for 1.3% of prostate carcinomas [4]. No consensus guidelines exist for the treatment of IDC-P specifically; the 2024 National Comprehensive Cancer Network (NCCN) guidelines for prostate cancer acknowledge the potential for IDC-P to be associated with higher risk disease and poorer outcomes, but do not provide treatment guidelines specific to IDC-P [5].

Preliminary investigation of the prognostic factors associated with this disease has been conducted to a limited extent. Dinerman et al. report on the incidence and survival of patients diagnosed with IDC-P between 2004 and 2013 found in the Surveillance, Epidemiology, and End Results (SEER) Program, ultimately finding that IDC-P was associated with various prognostic factors such as increased disease grade, positive surgical margins, etc., which were associated with increased cause-specific mortality relative to other prostate cancers [6]. Cui et al. created a nomogram for predicting both overall and cause-specific survival based on a SEER data for patients diagnosed with IDC-P based on various demographic, disease, and treatment variables [7]. This analysis demonstrated association between marital status, disease summary stage, grade, and the presence of metastasis and survival. To date, no analysis of IDC-P has been conducted in the context of the NCCN guidelines for prostate cancer and thorough subanalysis of current treatment recommendations (radical prostatectomy ± adjuvant radiotherapy or unimodal external beam radiotherapy (EBRT)) has yet to be executed. We seek to address this gap in the literature and provide a foundation for future investigation in the treatment of IDC-P.

## Materials and methods

### Data source and patient selection

The Surveillance, Epidemiology, and End Results (SEER) Program was queried to identify patients with a diagnosis of intraductal carcinoma of the prostate (IDC-P). Specifically, the 2000–2020 Incidence 17-registry data set was queried via a case listing session in SEER*Stat [8]. Inclusion criteria was “Site and Morphology: Site recode ICD-O-3/WHO 2008” = “Prostate”, “Histology recode—broad groupings” = “8500–8549: ductal and lobular neoplasms”, “First malignant primary indicator” = “Yes” (corresponding to a primary diagnosis of IDC-P), and ICD-O-3 Behavior Code equal to “Malignant.” Exclusion criteria were incomplete cause of death (COD)/survival/follow-up data and incomplete or unknown demographic and treatment data.

### Variable recoding/outcome data and analysis

Patient demographics, disease stage, treatment, and survival/COD information were acquired. Disease grade was not assessed given guidance by the International Society of Urological Pathology (ISUP) guidelines to avoid grading pure IDC-P [9, 10]. Definitive surgery was considered surgery with curative intent and included radical or total prostatectomy and pelvic exenteration, while local treatments such as laser ablation and transurethral resection of the prostate (TURP) were not. Data were analyzed in SPSS (Version 29.0; Armonk, NY: IBM Corp.), with a *p*-value of < 0.05 as the cutoff for statistical significance. 95% confidence intervals (CI) are reported in brackets. Chi-Square and Fisher exact tests are reported with exact two-sided *p*-values. Log-rank and Cox regression analyses were used to evaluate patient five-year overall survival (5y OS). Univariate analysis via Student’s t-test and hazard ratio (HR) calculation was also performed in Microsoft Excel (Version 2401; Redmond, WA; Microsoft, Inc.).

## Results

### Cohort determination and general information

**Fig. 1:**
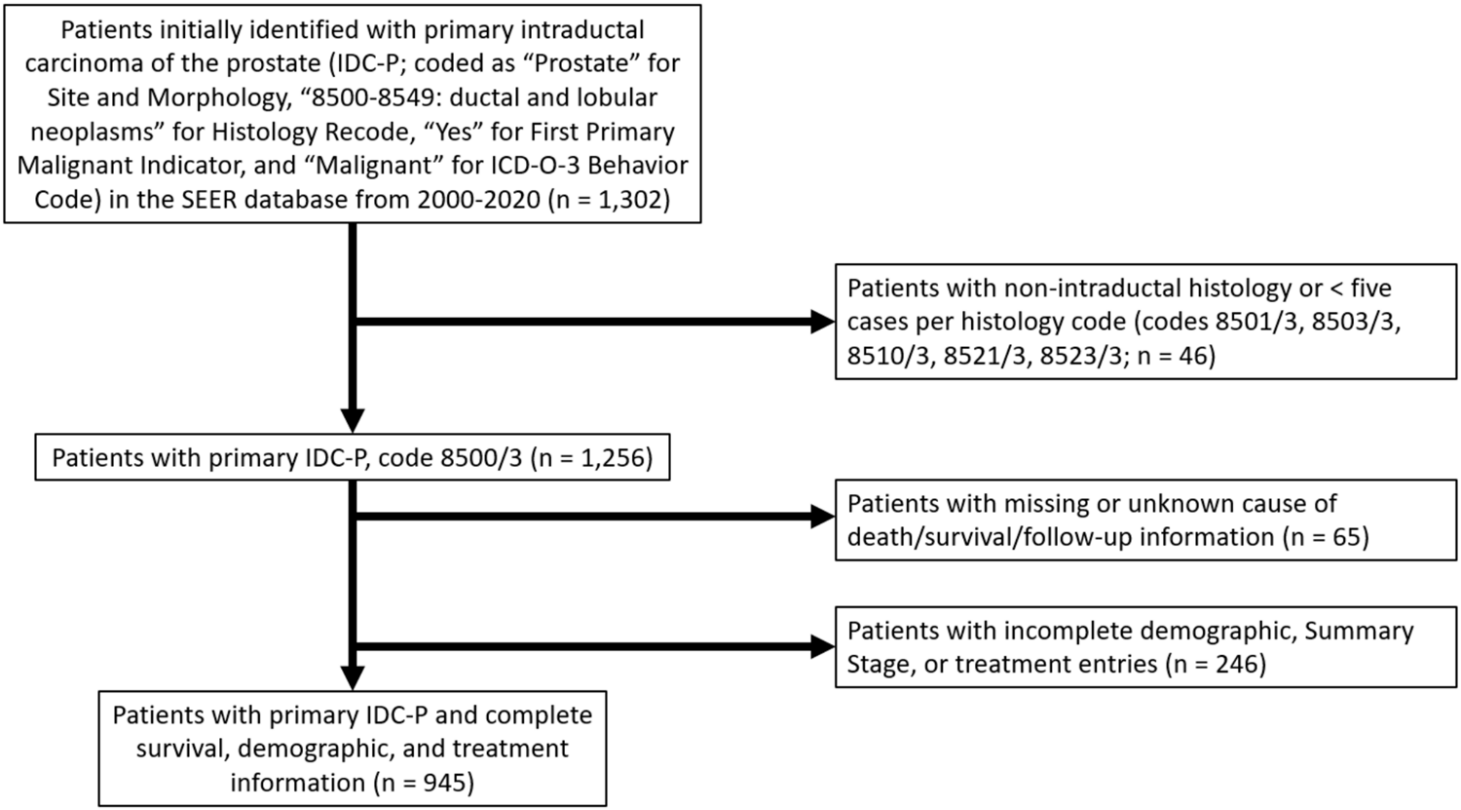
Selection of Patients with Primary Intraductal Carcinoma of the Prostate (IDC-P) from the SEER Database. The flowchart depicts the parameters used to identify patients with IDC-P for analysis. Patients were diagnosed with IDC-P between 2000 and 2020; all patients had histologically-confirmed disease

An initial cohort of 1302 patients with ductal or lobular neoplasms of the prostate were identified. This group was subsequently screened to remove cases with inapplicable disease histology or less than five cases (8501/3: comedocarcinoma, 8503/3: intraductal papillary adenocarcinoma, 8510/3: medullary carcinoma, 8521/3: infiltrating ductular carcinoma, and 8523/3: infiltrating duct mixed with other types of carcinoma) with one histology code retained: 8500/3: infiltrating duct carcinoma, not otherwise specified (NOS). This screening process and overall data parsing process can be found in Fig. 1.

### Cohort characteristics

Demographic and disease characteristics for the 945 patients identified with IDC-P can be found in Table 1. All patients were male. Average survival was 5.8 years (standard deviation = 4.8 years; median = 4.3 years). All patients had positive histology for IDC-P.

**Table 1:** Demographic and disease characteristics for patients with IDC-P identified in the SEER database, diagnosed between 2000 and 2020

Variable	Number (% of Cohort; *n* = 945)
Age at diagnosis	
< 65 years	355 (37.6%)
≥ 65 years	590 (62.4%)
Race	
American Indian	2 (0.2%)
Asian/Pacific Islander	87 (9.2%)
Black	108 (11.4%)
White	748 (79.2%)
Summary stage	
Local	479 (50.7%)
Regional	347 (36.7%)
Distant	119 (12.6%)

All patients identified were male

Treatment characteristics regarding modality, specific technique, and lymph node involvement can be found in Table 2. Four patients treated with chemoradiation and two patients treated with a radical prostatectomy with adjuvant radiotherapy and chemotherapy were identified.

**Table 2:** Treatment characteristics for patients with IDC-P identified in the SEER database, diagnosed between 2000 and 2020

Variable	Number (% of Cohort; n = 945)
Definitive surgery performed?*	
Yes	511 (54.1%)
No	434 (45.9%)
Surgery performed	
Pelvic exenteration	6 (0.6%)
Laser ablation	2 (0.2%)
No surgery	252 (26.7%)
Not specified	3 (0.3%)
Radical prostatectomy	499 (52.8%)
Subtotal prostatectomy	6 (0.6%)
TURP (with or without concurrent Laser Ablation)	117 (18.1%)
Unimodal surgical treatment	
Yes	434 (45.9%)
No	511 (54.1%)
Number of lymph nodes surgically removed	
None	507 (53.7%)
1–3	94 (9.9%)
≥ 4	280 (29.6%)
Unknown	64 (6.8%)
Unimodal radiotherapy (RT) treatment	
Yes	134 (14.2%)
No	811 (85.8%)
Type of RT administered	
Brachytherapy	12 (1.3%)
External beam RT (EBRT)	239 (25.3%)
EBRT with either brachytherapy or radioisotopes	17 (1.8%)
Radioisotopes	3 (0.3%)
No RT administered	674 (71.3%)
Chemotherapy administered	
Yes	23 (2.4%)
No/unknown	922 (97.6%)

Note that the SEER database codes the chemotherapy variable as either “Yes” or “No/Unknown;” more information is not available regarding chemotherapy

^*^Definitive surgery is defined as either radical or total prostatectomy, or pelvic exenteration

### Multivariate analysis

The results of multivariate Cox regression analysis with respect to patient survival can be found in Table 3. Both five- and ten-year overall and cause-specific survival were assessed (OS and CSS, respectively). It can be seen among patients for which CSS was assessed, age < 65 years at diagnosis was associated with increased 5-year CSS (HR = 0.65, *p* = 0.053) but did not impact 10y CSS (*p* = 0.11). However, age < 65 years at diagnosis was associated with increased OS for both 5-year and 10-year intervals (HR = 0.60, *p* = 0.009; HR = 0.54, *p* < 0.001, respectively).

**Table 3:** Cox Regression analyses pertaining to 5- and 10-year cause-specific survival (CSS) and overall survival (OS) for patients diagnosed with IDC-P between 2000 and 2020

Variable	Cause-specific survival (CSS)				Overall survival (OS)			
	Five-year (5y)		Ten-year (10y)		5y		10y	
	Hazard Ratio (HR)	*p*-value	HR	p-value	HR	*p*-value	HR	*p*-value
Age > = 65 / < 65 years	0.65	0.053	0.74	0.11	0.60	0.009	0.54	< 0.001
Race	N/A	0.43	N/A	0.24	N/A	0.99	N/A	0.32
Summary stage	N/A	< 0.001	N/A	< 0.001	N/A	< 0.001	N/A	< 0.001
Distant / (Local & Regional)	4.29	< 0.001	3.13	< 0.001	2.84	< 0.001	2.16	< 0.001
Local / (Regional & Distant)	0.46	0.010	0.46	0.002	0.66	0.065	0.70	0.045
Definitive surgery Y/N	1.70	0.37	2.69	0.051	2.50	0.064	4.11	< 0.001
Treatment only with surgery Y/N	3.29	0.033	3.70	0.005	2.32	0.08	1.88	0.085
Treatment with radiotherapy (RT) only Y/N	1.49	0.35	1.61	0.20	2.14	0.025	2.16	0.005
Type of RT	N/A	0.95	N/A	0.89	N/A	0.94	N/A	0.95
Chemotherapy Y/N	0.74	0.41	0.83	0.60	0.67	0.23	0.71	0.30
Regional lymph nodes surgically removed	N/A	0.66	N/A	0.58	N/A	0.25	N/A	0.60

Bivariate categorical variables have a HR listed, while variables with more than two categories have the overall *p*-value listed. Type RT has the following categories: Brachytherapy, External Beam Radiotherapy (EBRT), Radioisotopes, and Combination of EBRT and either Brachytherapy or Radioisotopes (based on coding in the SEER database). Definitive surgery was considered surgery with curative intent and included radical or total prostatectomy and pelvic exenteration, while local treatments such as laser ablation and transurethral resection of the prostate (TURP) were not

Increasing severity of summary stage was associated with worsened CSS and OS. Those with distant disease fared worse among all analyses, while patients with local disease did see improved survival (this only approached significance for 5-year OS: HR = 0.66, *p* = 0.065). Treatment with definitive surgery was associated with decreased survival, more strongly among 10-year survival analyses than 5 years. Similarly, patients treated with surgery-only or RT-only demonstrated decreased survival.

Race, number of lymph nodes surgically removed, treatment with chemotherapy (in conjunction with other treatments or as standalone therapy), and modality of RT treatment did not impact OS or CSS for any analysis.

### Radical prostatectomy (RP) ± adjuvant EBRT vs external beam radiotherapy (EBRT)

Stage-stratified survival analysis of patients treated with either radical prostatectomy (± adjuvant RT) or solely RT can be found in Table 4. Thirteen patients with distant disease were identified: 11 that received EBRT only, and one each for RP and RP with adjuvant EBRT. These patients have been excluded from this analysis. No difference in 5-year survival was seen among any of the treatments assessed for patients with local disease. Among patients with regional disease, 5y CSS among those with treated with EBRT only (71%) was inferior to both RP and RP with adjuvant EBRT (95% and 89%, respectively; *p* = 0.004). 5-Year OS analysis demonstrated a similar trend (p = 0.019). No difference was seen between RP and RP with adjuvant RT for 5-year OS (local: 97% vs 100%, *p* = 0.61; regional: 93% vs 86%; *p* = 0.23) or 5-year CSS (local: 99% vs 100%, *p* = 0.75; regional: 95% vs 89%; *p* = 0.22).

**Table 4:** Survival analysis for patients with intraductal carcinoma of the prostate (IDC-P) diagnosed between 2000 and 2020 undergoing radical prostatectomy (RP) ± adjuvant radiotherapy or EBRT only

Treatment	Summary stage (count)	Five-year survival [95% CI]		Ten-year survival [95% CI]	
		Cause-specific	Overall	Cause-specific	Overall
RP	Local (216)	99% [99%,101%]	97% [94%,100%]	98% [95%,100%]	90% [85%,96%]
	Regional (216)	95% [92%,99%]	93% [89%,97%]	89% [83%,95%]	77% [68%,86%]
RP + Adjuvant RT	Local (10)	100%	100%	100%	80% [44%,116%]
	Regional (49)	89% [78%,99%]	86% [75%,98%]	69% [51%,87%]	67% [49%,85%]
EBRT-only	Local (74)	100%	92% [85%,100%]	85% [68%,102%]	57% [39%,76%]
	Regional (20)	71% [46%,96%]	71% [46%,96%]	71% [46%,96%]	71% [46%,96%]

Patients with distant disease were excluded from this analysis. Further investigation into the survival for those with regional disease treated solely with EBRT, no patients died between 5 and 10 years after diagnosis, resulting in the same survival (CSS and OS) being calculated

Concerning 10-year survival, no difference was seen among treatment modalities for those with local disease when CSS was assessed (*p* = 0.12). However, patients with regional disease that underwent solely RP had increased 10-year CSS compared to both those that underwent RP with adjuvant EBRT and those that were treated with EBRT only (89% vs 69% and 71%, respectively; *p* = 0.007). However, when the same comparison was analyzed with respect to 10-year OS, no difference was seen among treatment modalities among those with regional disease (*p* = 0.20). On the other hand, those with local disease had decreased survival when treated solely with EBRT (10y OS = 57%) compared to RP and RP with adjuvant EBRT (90% and 80%, respectively; p < 0.001). No difference was seen between RP and RP with adjuvant EBRT (*p* = 0.58).

### Age vs treatment survival

**Fig. 2:**
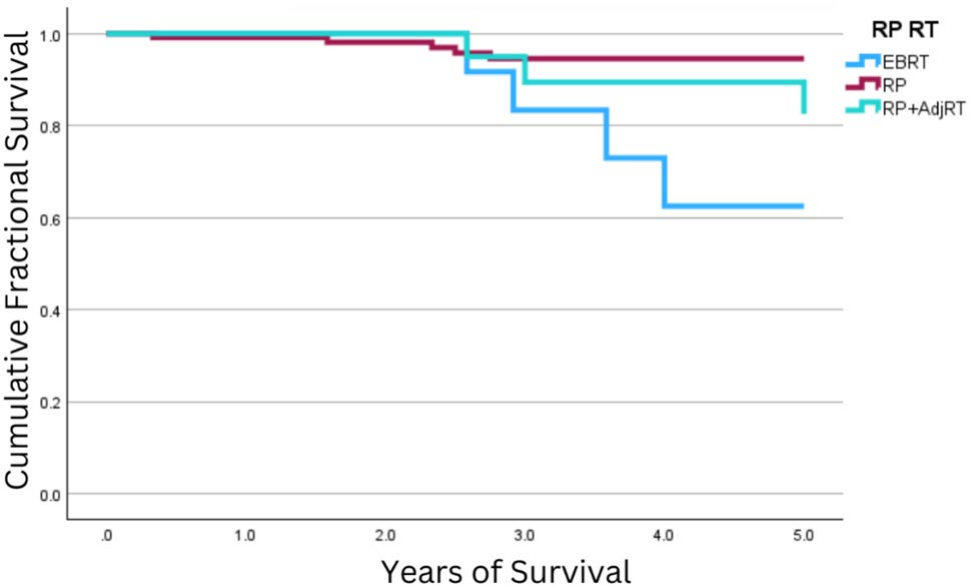
Five-year Overall Survival (5y OS) Among Patients ≥ 65 Years with Regional IDC-P. The Kaplan–Meier curve depicts 5y OS among patients diagnosed with intraductal carcinoma of the prostate (IDC-P), both ≥ 65 years at diagnosis and with regional disease. Patients treated with external beam radiotherapy (EBRT)-only had decreased 5y OS (63% [32%, 93%]) compared to both treatment with radical prostatectomy (RP; 92% [86%,98%]) and RP with adjuvant RT (77% [57%,98%]; *p* = 0.010)

Stratifying patients by summary stage and age < 65 years vs age ≥ 65 years at diagnosis can be found in Table 5. Regarding 5-year survival, patients with local or regional disease < 65 years at diagnosis had no difference in survival between treatment with RP, RP with adjuvant EBRT, or EBRT solely (OS: local disease: *p* = 0.77, regional disease: *p* = 0.91; CSS: local disease: *p* = 0.92, regional disease = 0.92). Similarly, those ≥ 65 years at diagnosis with local IDC-P saw no difference among treatment modalities (OS: *p* = 0.32; CSS: *p* = 0.72). However, among those ≥ 65 years with regional disease, treatment with EBRT-alone was associated with decreased survival (OS: *p* = 0.026; CSS: p = 0.010; see Fig. 2). It should be noted that eight patients < 65 years at diagnosis were treated with RP with adjuvant EBRT for local disease and two patients < 65 years at diagnosis were treated with EBRT-only for regional disease. Furthermore, only two patients ≥ 65 years at diagnosis were treated with RP with adjuvant EBRT for local disease.

**Table 5:** Survival analysis for patients with intraductal carcinoma of the prostate (IDC-P) diagnosed between 2000 and 2020 undergoing radical prostatectomy (RP) ± adjuvant radiotherapy or EBRT only, stratified by age

Age	Treatment	Summary stage (count)	Five-year survival [95% CI]		Ten-year survival [95% CI]	
			Cause-specific	overall	Cause-specific	Overall
Age < 65 years at diagnosis	RP	Local (122)	99% [98%,101%]	97% [93%,100%]	98% [94%,101%]	94% [89%,99%]
		Regional (100)	96% [91%,101%]	99% [97%,101%]	88% [78%,98%]	87% [76%,97%]
	RP + Adjuvant RT	Local (7)	100%	100%	100%	100%
		Regional (27)	95% [85%,105%]	95% [85%,105%]	68% [43%,92%]	68% [43%,92%]
	EBRT-only	Local (11)	100%	100%	75% [32%,118%]	60% [16%,104%]
		Regional (2)	100%	100%	100%	100%
Age ≥ 65 years at diagnosis	RP	Local (94)	98% [95%,102%]	97% [93%,101%]	98% [95%,102%]	84% [72%,95%]
		Regional (116)	95% [90%,99%]	92% [86%,98%]	91% [84%,98%]	68% [55%,82%]
	RP + Adjuvant RT	Local (2)	100%	100%	100%	50% [-21%,85%]
		Regional (22)	83% [64%,101%]	77% [57%,98%]	73% [49%,97%]	69% [45%,93%]
	EBRT-only	Local (63)	100%	91% [81%,100%]	90% [76%,104%]	58% [38%,78%]
		Regional (17)	100% [32%,93%]	63% [32%,93%]	63% [32%,93%]	63% [32%,93%]

Patients with distant disease were excluded from this analysis. Further investigation into the survival for those with regional disease treated solely with EBRT, no patients died between five and ten years after diagnosis, resulting in the same survival (CSS and OS) being calculated

The same trend was seen regarding 10-year survival analysis. Patients with local or regional disease < 65 years at diagnosis had no difference in survival between treatment with RP, RP with adjuvant EBRT, or EBRT solely (OS: local disease: *p* = 0.08, regional disease: *p* = 0.18; CSS: local disease: *p* = 0.18, regional disease = 0.12). Patients ≥ 65 years at diagnosis with local IDC-P had no difference in 10y CSS (p = 0.49), though those with regional disease had decreased survival when treated with EBRT alone (*p* = 0.025). On the other hand, patients ≥ 65 years at diagnosis with local disease saw decreased 10-year OS with treatment with EBRT alone (*p* = 0.022) and no difference was seen among those ≥ 65 years with regional disease, regardless of treatment modality (*p* = 0.48).

## Discussion

Intraductal carcinoma of the prostate (IDC-P) is a rare neoplasm, with a reported incidence of 3% among prostate biopsies [1]; one previous SEER analysis of 159,777 patients that underwent radical prostatectomy (RP) reported an incidence of 0.002% among this cohort [6]. Thus far the rarity of IDC-P has limited analysis of this disease at the population-level, with previous studies assessing demographic and treatment variables among 200–300 patients with IDC-P [6, 7]. To date, this analysis is the largest collection of patients diagnosed with IDC-P (*n* = 945) and the first to conduct such analysis in the context of the 2024 NCCN® guidelines [5].

Cox regression analysis of patients with IDC-P demonstrated increased survival among patients < 65 years at diagnosis, though this was limited to both 5- and 10-year OS (*p* = 0.009 and < 0.001, respectively). In the context of what has been reported as an aggressive disease [2], this may be the result of younger patients having greater OS than those that are older, especially given that no difference in CSS was noted at either 5- or 10-year. Summary Stage, on the other hand, was significant across all timeframes and for both OS and CSS. This appears to be driven more by the decreased survival associated with patients with distant disease (*p* < 0.001 across all timeframes and survival analysis type), though local disease compared to regional and distant was nonsignificant only concerning 5-year OS (*p* = 0.065). This result is also in keeping with the results put forth by Cui et al. in a previous SEER analysis [7]. In contrast though, patient age was a factor concerning analysis of both five- and 10y OS, while patient age was not a significant factor presented in Cui et al.; this may have been secondary to the smaller cohort (*n* = 280) for this paper.

Similarly, no consistent survival benefit was noted with Cox regression concerning any modality of oncologic treatment, and in some cases (e.g. 10y OS for treatment with surgery only: hazard ratio (HR) = 4.11, *p* < 0.001; treatment with radiotherapy (RT) only: HR = 2.16, *p* = 0.005) treatment was associated with decreased survival. This may be in the context of aggressive disease but initially the authors considered that this could also indicate under-treatment of disease with unimodal rather than multimodal therapy; current NCCN guidelines stratify guidance by patient risk group, but in general allow for either RT or RP as the initial treatment for patients with > 10 years of expected survival (this number decreases to 5 years among those with higher-risk disease) [5]. Additionally, no difference was seen between types of RT, treatment with chemotherapy, or concerning the number of lymph nodes surgically removed during treatment.

However, stage-stratified analysis of patient survival further stratified by treatment modality demonstrated decreased survival among patients undergoing RT as opposed to surgery or surgery with adjuvant RT. While no difference in 5-year survival was seen between any treatment modality among those with local disease, those treated with RT for regional IDC-P had both decreased 5-year OS and CSS vs RP alone and RP with adjuvant RT (*p* = 0.019 and 0.004, respectively). 10-Year survival analysis results were somewhat mixed, but in general external beam RT (EBRT) was associated with decreased survival. Across all timeframes RP alone was non-inferior to RP with adjuvant RT, though patients treated with RP alone made up a much greater proportion of the entire cohort compared to those treated with adjuvant RT (45.7% vs 6.2%).

Further analysis based on patient age (< 65 years vs ≥ 65 years at diagnosis) demonstrated no difference in 5- or 10-year survival among patients < 65 years with respect to treatment modality. Among patients ≥ 65 years with regional disease though, treatment with EBRT-alone was associated with decreased survival (OS: *p* = 0.026; CSS: *p* = 0.010). Furthermore, patients ≥ 65 years at diagnosis with local disease saw decreased 10y OS with treatment with EBRT alone (*p* = 0.022). In the context of these results (both in general and stratified by age), treatment of IDC-P with RT may favor younger patients, both from the standpoint of noninferior survival outcomes and from the decreased comorbidities associated with RT vs surgery [11]. However, among patients ≥ 65 years at diagnosis that can tolerate surgery, RP alone may prove sufficient and provide improved outcomes with fewer side effects as compared to wither EBRT alone or RP with adjuvant RT. In general RP alone was found to be noninferior to RP with adjuvant RT; in the context of this trend and the lack of impact chemotherapy and surgical lymph node removal had on survival with Cox regression analysis, it may be the relegation of IDC-P to the prostatic ducts that limits the benefits of adjuvant RT and systemic treatment [12].

This analysis was limited in some regards. As a retrospective, population-level analysis, the granularity of the data was limited and subject to the inherent bias of such studies. Regarding the limited granularity, no information on the RT dose, type of chemotherapy, or if treatment was conducted with palliative intent was available. Additionally, information on patient frailty or the presence of comorbidities, factors which likely influenced the decision to proceed with surgery versus RT, were not available. This analysis did not incorporate analysis or stratification based on prostate serum antigen (PSA) values, which are commonly included in the surveillance of other types of prostate cancer. We do believe future analysis of IDC-P may benefit from exploration of these values. Finally, information on the exact stage of IDC-P is limited in this analysis, as the authors have used summary stage in its place. This was done to address the issue of changing definitions of prostate cancer staging and was also used in light of how this cancer has been treated historically.

## Conclusions

Patients < 65 years at diagnosis with intraductal carcinoma of the prostate, a rare prostatic neoplasm, may benefit from radiotherapy versus radical prostatectomy (RP), though RP remains the treatment associated with the highest survival among patients ≥ 65 years concerning both overall and cause-specific survival. RP alone was noninferior to RP with adjuvant radiotherapy in this analysis. These results should be taken in the context of current prostate cancer treatment guidelines, and further research into the risk stratification and optimal treatment of these patients is warranted.

## Data Availability

The data that support the findings of this study are publicly available from https://seer.cancer.gov/.
