# Correction: Treatment outcomes and comparative survival analysis of intraductal carcinoma of the prostate

**DOI:** 10.1007/s11255-025-04774-x

**Published:** 2025-09-04

**Authors:** Taylor Stamey, Kristen Armel, Andrew W. Ju, Shoujun Chen, Musharraf Navaid, Arjun Bhatt, Michael C. Larkins

**Affiliations:** 1 Brody School of Medicine (BSOM), East Carolina University (ECU), 600 Moye Blvd, Greenville, NC 27834 USA; 2 Department of Radiation Oncology, ECU, Greenville, NC USA; 3 Department of Pathology & Laboratory Medicine, ECU, Greenville, NC USA; 4 Division of Hematology/Oncology, Department of Internal Medicine, ECU, Greenville, NC USA; 5 Department of Emergency Medicine, Boonshoft School of Medicine at Wright State University, 2555 University Blvd, Fairborn, OH USA

**Correction: International Urology and Nephrology** 10.1007/s11255-025-04485-3

The disclaimer statement was missing from this article and should have read as given below.

Disclaimer: “The views and opinions expressed in this article/presentation are those of the author(s) and do not necessarily reflect official policy or position of the United States Air Force, Defense Health Agency, Department of Defense, or U.S. Government.”

The original article has been corrected.

